# Climate drives adaptive genetic responses associated with survival in big sagebrush (*Artemisia tridentata*)

**DOI:** 10.1111/eva.12440

**Published:** 2017-03-03

**Authors:** Lindsay Chaney, Bryce A. Richardson, Matthew J. Germino

**Affiliations:** ^1^ Plant and Wildlife SciencesBrigham Young UniversityProvoUTUSA; ^2^ USDA Forest ServiceRocky Mountain Research StationProvoUTUSA; ^3^ U.S. Geological SurveyForest and Rangeland Ecosystem Science CenterBoiseIDUSA; ^4^ Present address: Department of BiologySnow CollegeEphraimUTUSA

**Keywords:** adaptation, atmospheric decoupling, cold adaptations, minimum temperatures, polyploidy, population differentiation, survival analysis

## Abstract

A genecological approach was used to explore genetic variation for survival in *Artemisia tridentata* (big sagebrush). *Artemisia tridentata* is a widespread and foundational shrub species in western North America. This species has become extremely fragmented, to the detriment of dependent wildlife, and efforts to restore it are now a land management priority. Common‐garden experiments were established at three sites with seedlings from 55 source‐populations. Populations included each of the three predominant subspecies, and cytotype variations. Survival was monitored for 5 years to assess differences in survival between gardens and populations. We found evidence of adaptive genetic variation for survival. Survival within gardens differed by source‐population and a substantial proportion of this variation was explained by seed climate of origin. Plants from areas with the coldest winters had the highest levels of survival, while populations from warmer and drier sites had the lowest levels of survival. Survival was lowest, 36%, in the garden that was prone to the lowest minimum temperatures. These results suggest the importance of climatic driven genetic differences and their effect on survival. Understanding how genetic variation is arrayed across the landscape, and its association with climate can greatly enhance the success of restoration and conservation.

## 1 Introduction

Adaptive differentiation among populations within a species range is well documented. Examples date back to the classical studies of Turesson (1925) and Clausen, Keck, & Hiesey (1940) and continue into recent literature (Anderson, Lee, Rushworth, Colautti, & Mitchell‐Olds, 2013; Bischoff et al., 2006; Galen, Shore, & Deyoe, 1991; Galloway & Fenster, 2000; Hereford, 2009; Kawecki & Ebert, 2004; Leimu & Fischer, 2008; Pratt & Mooney, 2013; Vergeer & Kunin, 2013). In species with large geographic ranges, it is common to have populations that occupy different climatic and ecological conditions. Divergent selection can subsequently result in the evolution of phenotypic traits that are better adapted to their different habitats (Chevin & Lande, 2011; Kawecki & Ebert, 2004). Climate variation is a strong selective agent that leads to population differentiation (Jump & Peñuelas, 2005; McKay et al., 2008; R. C. Johnson, Cashman, & Vance‐Borland, 2012). While climatic factors largely determine a species range (Sexton, McIntyre, Angert, & Rice, 2009), climatic differences can also stimulate local adaptation, population divergence, and, in some cases, speciation (Hua & Wiens, 2013).

Genecological studies (sensu Turesson, 1923) are a primary means for assessing how landscape and spatial variation causes genetic differences among plant populations. For example, cold temperatures have been found to be an important factor affecting adaptation in many temperate plant species, influencing how and when plants grow to maximize water availability or avoid freezing (Richardson et al., 2014; St. Clair, Mandel, & Vance‐Borland, 2005). Responses to the environment can then be used to create geographically delineated seed zones to help ensure that seed can be transferred among areas of similar climate, increasing odds of appropriately adapted vegetation and successful restoration (Bower, St. Clair, & Erickson, 2014). Recent genecology studies have found that winter minimum temperatures are a primary agent of genetic variation in conifers (Rehfeldt, 2004; Rehfeldt & Jaquish, 2010; Rehfeldt, Jaquish, Sáenz‐Romero, et al., 2014), native grasses (R. C. Johnson et al., 2012; St. Clair, Kilkenny, Johnson, Shaw, & Weaver, 2013), and shrubs (Horning, McGovern, Darris, Mandel, & Johnson, 2010; Richardson et al., 2014). However, sufficient information on climatic adaptations is lacking in the majority of native grasses, forbs, and shrubs used for restoration purposes.

*Artemisia tridentata* (big sagebrush) is a widespread desert shrub that dominates much of western North America. Three predominant subspecies of *A. tridentata* are defined by ecological niches affected principally by climate, soil type, and soil depth (McArthur, 1994). Common gardens or variation in subspecies habitats has revealed genetic divergence across local gradients, such as across hillslopes or topographic positions (Graham, Freeman, & McArthur, 1995; Wang, McArthur, & Freeman, 1999; Wang, McArthur, Sanderson, Graham, & Freeman, 1997) that can be at least partly explained by differences in climate (Kolb & Sperry, 1999). Local adaptation within (Brabec et al., 2015) and among (Brabec, Germino, & Richardson, 2016) subspecies of *A. tridentata* was evident from survival analysis of a small number of populations (four and 11, respectively). These considerations and previous findings suggest a high likelihood of local climate adaptation in big sagebrush, although a comprehensive, range‐wide assessment is needed. As a foundational species in the Great Basin ecosystems (Prevéy, Germino, Huntly, & Inouye, 2010), it is host to sagebrush‐obligate wildlife, such as the greater sage‐grouse (*Centrocercus urophansianus*), and is of conservation concern (Knick & Connelly, 2011). Millions of hectares of sagebrush have been eliminated during the past century due to change in land use (e.g.*,* grazing), exotic plant invasion, and increased frequency of wildfire (Miller et al., 2011). Restoration of *A. tridentata* ecosystems through seeding or planting has occurred on millions of hectares in the western United States in recent decades and continues to be a management priority. Restoration success of sagebrush is contingent on a number of factors including weather, soils, seed mix composition, and seeding and planting techniques (Hardegree et al., 2011). Genetic adaptation, however, is by far the most critical factor for long‐term resiliency of these ecosystems, underscoring the need to better understand how climate affects genetic variation in this species.

In the current study, we assess adaptive genetic variation within and between 55 source‐populations of *A. tridentata* using common‐garden experiments at three sites. The use of common gardens allows researchers to test the genetic basis for phenotypic differences among a relatively large number of plant populations. In genecology studies, geospatial patterns of these genetic traits are then related to plant source climates using regression and bioclimatic modeling. In this study, we address two main questions: (i) How is survivorship influenced by genetics, environment and/or their interactions? (ii) What are the climate patterns that affect survivorship? These results will be fundamental in understanding adaptive genetic variation and fitness in *A. tridentata*, which are essential to restoration in a changing climate.

## 2 Methods

### 2.1 Source seed collection

*Artemisia tridentata* includes three predominant and widely recognized subspecies and two cytotype (ploidy) levels. Subspecies *tridentata* (basin big sagebrush) is diploid or tetraploid, grows in lower elevations with deep soil, and exhibits rapid growth and tall stature. Subspecies *wyomingensis* (Wyoming big sagebrush) is tetraploid, occurs in relatively warm and dry areas with shallow soils, and exhibits slow growth and a shorter stature. Subspecies *vaseyana* (mountain big sagebrush) is diploid or tetraploid, occurs in cooler and relatively mesic conditions at higher elevations, and has a compact growth form (McArthur & Sanderson, 1999). Tetraploids are polyphyletic, deriving from multiple events within and between subspecies (Richardson, Page, Bajgain, Sanderson, & Udall, 2012). Seeds from each of the three subspecies of *A. tridentata* were collected from 55 sites, hereafter referred to as populations, in the fall of 2009 (Table S1). A population typically consisted of half‐sib families collected from eight to ten parents. These seeds were collected at random over an area approximately 1 ha. Subspecies were identified by morphology (height and stature), ultraviolet iridescence (following the methods of McArthur, Welch, & Sanderson, 1988), cytotype, molecular genetic analyses (Richardson et al., 2012), and volatile organic compounds (Jaeger, Runyon, & Richardson, 2016). Cytotype was typically uniform within a source‐population, but could vary among subspecies (Table S1), with the exception of *wyomingensis* that is only tetraploid.

### 2.2 Experimental design

A five‐year common‐garden experiment was designed with three sites to understand how genetic and environmental factors contribute to *A. tridentata* survival (Table 1; Fig. S1). Garden sites were chosen based on the following criteria: (i) climates that were representative of each of the subspecies, (ii) adjacent to weather stations, and (iii) accessibility for data collection and maintenance. The garden in Ephraim, Utah, USA, is a relatively cold basin site with a dry climate typical of ssp. *tridentata;* the garden in Majors Flat, Utah, USA, is a higher‐elevation mountainous site with a mesic climate typical of ssp. *vaseyana*; and the garden in Orchard, Idaho, USA, is a warm plain site with a dry climate typical of ssp. *wyomingensis*. Weather data at the common gardens were obtained from adjacent weather stations (USDA, Natural Resources Conservation Service and Forest Service).

**Table 1:** Geographic and climatic attributes of the three common gardens

Garden	Latitude	Longitude	Elevation (m)	MTCM (oC)	MTWM (oC)	TDIFF (oC)	MAP (mm)	SMRP (mm)
Ephraim, Utah	39.369	−111.578	1690	−9.7	21.2	30.9	276	54
Majors Flat, Utah	39.339	−111.520	2105	−4.7	20.8	25.5	442	72
Orchard, Idaho	43.322	−115.998	974	−2.9	25.0	27.9	257	4

MTCM, mean temperature of the coldest month; MTWM, mean temperature of the warmest month; TDIFF, temperature difference (MTWM‐MTCM); MAP, mean annual precipitation; SMRP, summer precipitation (July and August).

Climate data are based on values from 2010 to 2013 water years.

Seeds were collected from 55 wild source‐populations found throughout the species distribution (Table S1; Fig. S1). After collection, seeds were cleaned and placed into a freezer until sowing. Replicate seeds from each population were sown into six‐inch cone‐tainers with a custom soil mix for native plants, established in a greenhouse for approximately 3 months, and cold hardened outside for 2 weeks. Due to differences in climate (Table 1), the Ephraim and Orchard gardens were planted in late April, and the Majors Flat garden was planted in early June 2010. The germination and planting dates for Majors Flat seedlings were delayed to ensure plants were approximately the same age when transplanted. Seedlings were planted in a completely randomized design in a lightly tilled plot that had an above and below‐ground fence to discourage ungulate and rabbit herbivores. Due to variation in seed yield and germination, populations in each garden ranged from 1 to 11 individuals with an average of 7.8 plants per population (Table S1). Plant spacing was 1.5 m between rows and 1 m within rows, and a border row was planted around the perimeter to minimize edge effects. To ensure establishment, plants were watered periodically during the first growing season in 2010.

### 2.3 Data collection

Phenotypic measurements focused on *A. tridentata* survival in the three gardens. A plant was considered dead when all foliage was missing and no re‐greening occurred during the growing season. Mortality for each experimental plant was recorded in each garden an average of 14.3 times during the experiment (approximately four census dates each year). A small proportion (<6%) of outplanted plants were excluded from the experiment because they did not survive the first growing season and thus could not address our main question of how climate affects sagebrush survival, leaving a total sample number of *n* = 1,299. The number of excluded and total number of plants at each garden are as follows: 10/459, Ephraim, 26/458, Majors Flat, 42/460, Orchard.

Climate for each source‐population was utilized to determine how source climate affects survival. Geographic coordinates and elevation for each source‐population were used to estimate climate variables from thin plate splines using ANUSPLIN v 4.1 (Hutchinson, 2000) based on normalized monthly means from years 1961 to 1990 with a 0.0083^o^ resolution (~1 km^2^) (Crookston & Rehfeldt, 2012; Rehfeldt, 2006). A total of 40 climate variables were examined for this study including 18 yearly and season temperature and precipitation indices and 22 interactions among different variables that can be important to plant adaptation (Table S2). This follows the methods of other similar ecological genetic studies (e.g., Bansal, Harrington, Gould, & St.Clair, 2015; Joyce & Rehfeldt, 2013; Ledig, Rehfeldt, Sáenz‐Romero, & Flores‐López, 2010; Rehfeldt, Jaquish, López‐Upton, et al., 2014; Richardson et al., 2014).

### 2.4 Data analysis

Data were first analyzed to assess significance of genetics, environment, and their interaction for survival in *A. tridentata* across the three common gardens. This analysis was performed using a generalized linear mixed model with a quasi‐binomial error distribution on survival proportion of each source‐population across the three gardens. A multivariate response variable with number of survivors and the number of dead for each source‐population was used to account for differences in sample sizes. Estimates of genetic effects were specified as fixed effects and included plant subspecies:cytotype group, and source‐population climate variables. Estimates of environment and genetics by environment were specified as random effects and included garden and the interaction of garden and subspecies:cytotype group. Model was fit using the *glmer* function from the *lme4* package (Bates, Mächler, Bolker, & Walker, 2015) with the objective to find the model with the highest predictive value, combined with the fewest number of climate variables. To do this, methods similar to R. C. Johnson et al. (2012) were used. More specifically, a forward selection procedure was used to select the source‐population climate variables that best explain variation for survival in the model and variables were no longer added when not significant (*p *>* *.05). Significance for each of the effects was calculated based on likelihood‐ratio chi‐square tests. Conditional and marginal *R* ^2^ values were calculated with the *rsquared.glmm* function (Nakagawa & Schielzeth, 2013); conditional *R* ^2^ describes the variation explained by both fixed and random effects; and a marginal *R* ^2^ describes fixed effects alone. To provide a more accurate model, only populations with more than two samples were evaluated, resulting in *n* = 152 populations among the three gardens. This model is also referred to as the genecological model and is used in the mapping discussed below.

Next, a more detailed survival analysis was performed to determine the rate of survival within the Ephraim garden. To do this, the *survival* package in R (Therneau & Lumley, 2014) was utilized to fit a parametric survival model. This method accounts for the interval and right‐censored nature of the data (i.e., the date of death is after some known date). Survival rate was determined to have a lognormal distribution, based on a lower AIC value and higher maximum likelihood of our statistical model. Kaplan–Meier estimators were used to generate survival curves for each subspecies:cytotype group. Differences in survival curves were tested using the log‐rank test, and post hoc pairwise comparisons were performed with a Bonferroni‐corrected confidence level for multiple comparisons. Median survival and probability of survival at different time intervals were calculated for both subspecies:cytotype group and source‐population. All data analyses were performed in the R statistical environment (v 3.2.1; R Core Team 2015). Analysis code and data are available for public access (scripts: https://github.com/lchaney/Sagebrush_Mort; data: http://dx.doi.org/10.5061/dryad.32s2t).

### 2.5 Climate mapping

A map was generated for the probability of survival utilizing the genetic effects of the generalized linear mixed model. Probability of survival was calculated for individual cell values, 0.00833^o^ (approximately 1 km^2^), using the *yaImpute* package in R (Crookston & Finley, 2007). The generalized linear mixed model was projected within the climatic niche boundaries of subspecies *wyomingensis* (Still & Richardson, 2015) using QGIS (QGIS Development Team 2015). This was completed using the log‐link transformed coefficients of the intercept and slopes of the fixed effects in the generalized linear mixed model (i.e., the slope of the two climate variables and *wyomingensis*). We focused on subspecies *wyomingensis* because it is the subspecies of greatest conservation concern and restoration investment. Further, distributions of subspecies *tridentata* and *wyomingensis* can often be sympatric at this spatial scale, with the distinction that *tridentata* is usually controlled by local topographic features such as soil depth and higher moisture features (e.g., fence lines, roadways, washes) that provide additional moisture and are difficult to model (Still & Richardson, 2015).

## 3 Results

Survival of *A. tridentata* varied in the three common gardens as a function of environment, genetics, and their interaction. Survival was significantly different between gardens, indicating an effect of environment on *A. tridentata* survival ($\chi_{1}^{2}$= 15.417, *p *<* *.001; Table 2). Survival at Ephraim was much lower than the other two gardens; 36% of *A. tridentata* at Ephraim survived compared to 85% and 78% at Majors Flat and Orchard, respectively (Fig. S2). The genetic influence on survival was environmentally dependent, as indicated by a significant subspecies:cytotype group by garden effect ($\chi_{1}^{2}$ = 14.107, *p *≤ .001; Table 2) which denotes differences in phenotypic plasticity by subspecies:cytotype group. This was expected considering each garden represented the habitat type for a different subspecies. Subspecies:cytotype group was a significant predictor of survival in *A. tridentata*, indicating a genetic component of survival ($\chi_{4}^{2}$ = 15.372, *p *=* *.004; Table 2). Evidence of genetic effects were further shown by population differentiation, specifically significant effects of climate of populations’ origins on their survival. Two source‐population climate variables, TDIFF (temperature difference between mean temperature in the coldest month and mean temperature in the warmest month) and marginally significant SMRP (summer precipitation, July and August), were found to best explain variation in survival ($\chi_{1}^{2}$ = 32.764, *p *<* *.001 and $\chi_{1}^{2}$ = 3.712, *p *=* *.054, TDIFF and SMRP, respectively; Table 2).

**Table 2:** Generalized linear mixed genecological model that explains effects of genetics, population climate of origin and subspecies:cytotype group, and environment, garden, on survival of *A. tridentata* across the three common gardens

	*df*	Chi‐square	*p*
Fixed effects			
TDIFF	1	32.764	<.001
SMRP	1	3.712	.054
Subspecies:cytotype	4	15.372	.004
Random effects			
Garden	1	15.417	<.001
Garden × subspecies:cytotype	1	14.107	<.001

TDIFF: temperature difference between mean temperature in the coldest month and mean temperature in the warmest month; SMRP: summer precipitation (July and August).

**Figure 1:**
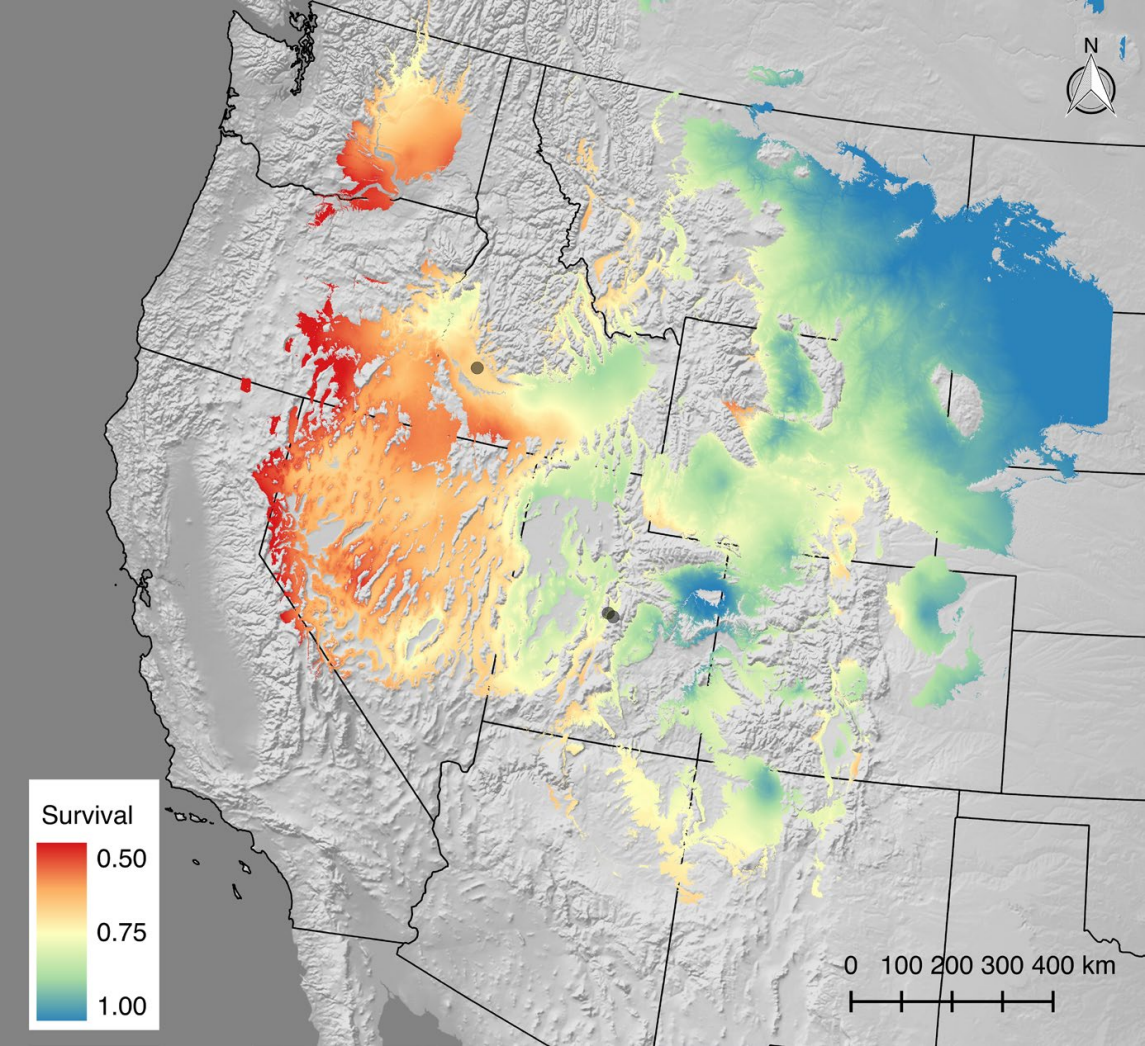
Genecological projection of survivorship in big sagebrush in three common gardens. Common‐garden locations shown as black circles. Log‐link transformed coefficients of slopes and intercepts of the generalized linear mixed model were used to project probability of survival into the niche boundaries of *wyomingnesis* big sagebrush (Still & Richardson, 2015). Climate predictors included in the model are TDIFF and SMRP. Areas that have greater temperature differences between summer and winter and wetter summers have higher probability of survival (blue), while areas with more moderated temperatures and drier summers have lower probability of survival (red). TDIFF: temperature difference between mean temperature in the coldest month and mean temperature in the warmest month; SMRP: summer precipitation (July and August). Hillshade background is based on the US Geological Survey Digital Elevation Model

Mapping the generalized linear mixed genecological model revealed a relationship of survival in the gardens to continental‐scale gradients in climate. Specifically, big sagebrush from regions with greater seasonal temperature differences and higher summer precipitation supported a greater probability of survival in the central range. These areas were predominantly found in interior regions of the continent with blue and green shades (Figure 1). In contrast, populations from climates that consisted of moderated winter temperatures and drier summers had lower probability of survival in the gardens, mapped in red and dark orange (Figure 1).

**Figure 2:**
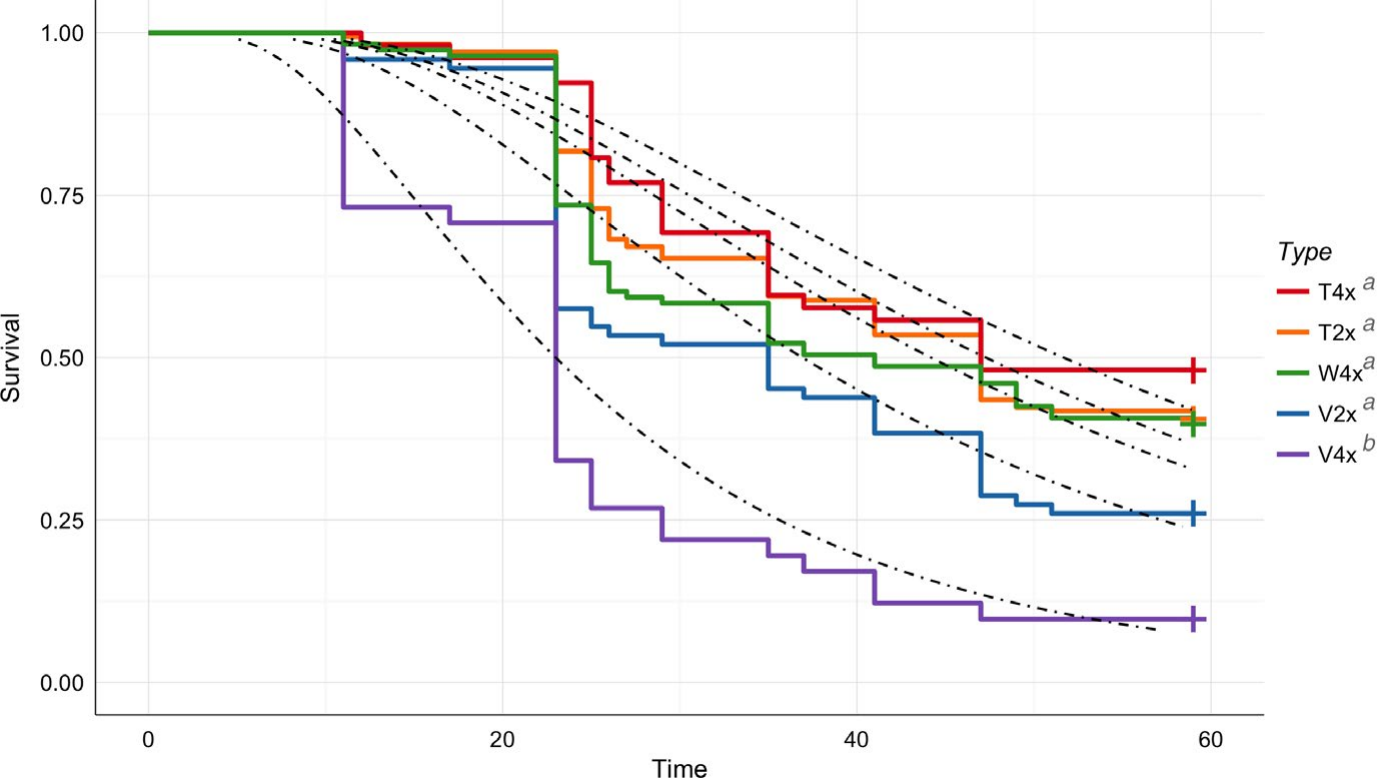
Kaplan–Meier survivorship curves by subspecies:cytotype group in big sagebrush at the Ephraim garden. Model estimates of the log‐normal survivorship curve from the survivorship regression are overlaid to assess fit of model. Survivorship was significantly different by subspecies:cytotype group. Letters indicate significant difference from Bonferroni post hoc comparisons

**Figure 3:**
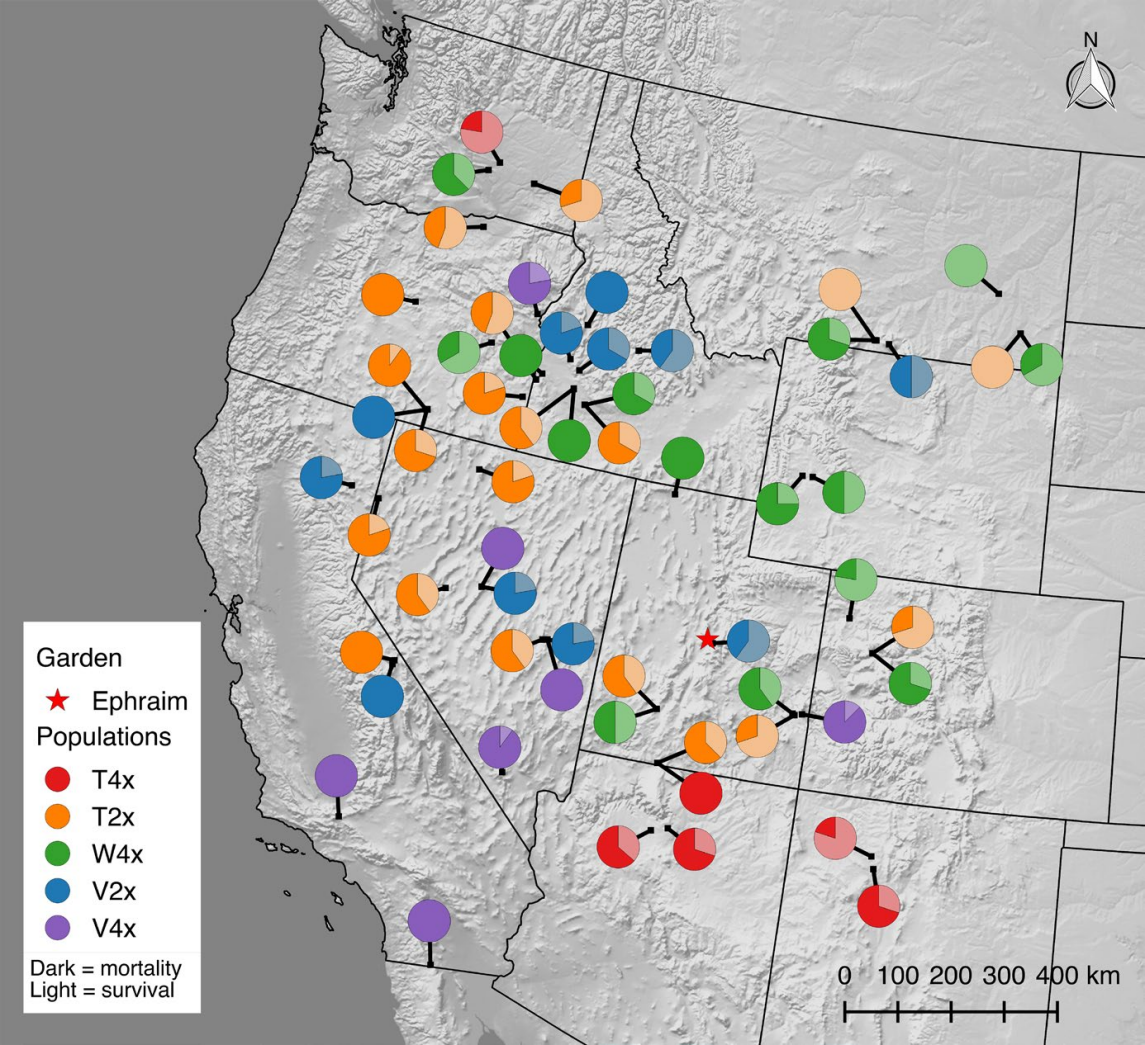
Proportion survival at the Ephraim garden for each source‐population. The pie charts show the proportion of survival for each population, dark shade indicates mortality, while the light shade indicates survival. Color of each population is determined by subspecies:cytotype group. Hillshade background is based on the US Geological Survey Digital Elevation Model. T4x = tetraploid *tridentata*; T2x = diploid *tridentata*; W4x = tetraploid *wyomingensis*; V2x = diploid *vaseyana*; V4x = tetraploid *vaseyana*

To further investigate the low rate of survival at Ephraim, we performed a detailed survival analysis for this garden. Survival increased at a log‐normal rate with a scale of 0.649, that is, the rate of mortality decreased over time (Figure 2; Table S3). Rate of survival was significantly different for tetraploid *vaseyana* (V4x) than for the other subspecies:cytotype groups ($\chi_{4}^{2}$ = 52.6, *p *<* *.001). Specifically, survival time of tetraploid *vaseyana* (V4x) was 0.445 times shorter than the baseline tetraploid *tridentata* (T4x) (*e* ^−0.810^; Table S3). The median survival of tetraploid *vaseyana* (V4x) was 23 months (*i.e*., only a 50% chance of survival at 23 months), and there was less than 10% chance of survival at 48 months. In contrast, tetraploid *tridentata* (T4x) had a median survival of 47 months (Table S4). Survival varied considerably around the mean survival proportion of 0.65 among source‐populations at Ephraim (Figure 3, Table S5). Some populations experienced 100% mortality after the first winter (i.e., population CAV4), while all families of other populations persisted throughout the experimental period (i.e., populations MTT1, MTW2).

## 4 Discussion

### 4.1 Adaptive genetic responses to climate

Growth in *A. tridentata* occurs primarily in the spring, while soil moisture levels are high (Germino & Reinhardt, 2014; Schlaepfer, Lauenroth, & Bradford, 2014), yet minimum temperatures are often physiologically limiting and even lethal (Brabec et al., 2016). Strong differences in avoidance and tolerance were evidence among seedlings of the subspecies:cytotype groups of *A. tridentata* related to their mortality and suggested the importance of freezing response as an axis for adaptive differentiation (Brabec et al., 2016). At the landscape level, selection pressures of different climates have potentially generated differences in phenology and growth strategies for *A. tridentata*. One hypothesis would be that interior regions, with a continental climate (greater summer–winter temperature extremes), support big sagebrush populations that have a growth strategy that confers resistance, tolerance, or avoidance of freezing temperatures through physiological mechanisms or deferment/delay of growth until after the spring frost period. In more moderate climates to the south and west, we further hypothesize that *A. tridentata* initiates growth earlier in spring to capture spring soil moisture, resulting in greater risk of exposure to freezing in the gardens we evaluated. Below, we discuss how results from our analyses support this hypothesis.

Results from the generalized linear mixed model indicated that source‐population climate is a strong predictor of survival in *A. tridentata*. The final generalized linear mixed genecological model explained 44% of variation (conditional *R* ^2^ = .439). The genetic effects of the final model, climate variables TDIFF and SMRP along with plant subspecies:cytotype group, explained 17% of the variation (marginal *R* ^2^ = .171). Survival patterns follow a southwest to northeast gradient where, in general, populations from higher latitudes and lower longitudes support higher survival (Figure 1). These continental regions, such as Montana and Colorado, have colder winters and have higher survival. These populations appear adapted to low minimum winter temperatures and later springs. Lower survival occurred in plants from areas to the southwest with more moderated climates. Growth in these regions likely begins earlier in the spring to maximize water availability from snowmelt and has low probability of freezing due to milder winters. However, early growth in Ephraim, where there was exposure to late winter extreme low minimum temperatures and more prevalent spring frosts (Fig. S3), puts the plant at risk for freezing and cold‐related drought (Lambrecht, Shattuck, & Loik, 2007).

Our results support previous research that also indicated the adaptive importance of minimum temperatures (Bower et al., 2014; Erickson, Mandel, & Sorensen, 2004; Horning et al., 2010; Johnson, Sorensen, Bradley St Clair, & Cronn, 2004; Richardson et al., 2014; St. Clair et al., 2013). Although *A. tridentata* is generally thought of as being cold tolerant, winterkill has been documented in natural populations (Walser et al., 1990) and common gardens of seedlings at relatively warm sites (Brabec et al., 2016). Other studies of *A. tridentata* also found differences in freeze tolerance associated with source location, such as greater freezing acclimation for seedlings from higher‐elevation seed sources (Loik & Redar, 2003).

While we found differences in survival rate among plant subspecies:cytotype groups, these differences were highly influenced by source‐population. Post hoc comparisons of our survival analysis found that only tetraploid *vaseyana* (V4x) was significantly different from other subspecies:cytotype groups. This may be attributable to source‐population origins rather than true taxonomical differences, considering how many tetraploid *vaseyana* (V4x) were from the southwest United States (e.g., CAV3, CAV4, NVV3). Previous work has suggested that *A. tridentata* subspecies are adapted to different habitats (Mahalovich & McArthur, 2004; McArthur, 1994; Wang et al., 1997), which can often be defined on local spatial scales. However, our findings suggest that within subspecies, populations are principally adapted to clines in continentality (Table 2).

### 4.2 Microclimate variation influences survival

Survival of *A. tridentata* differed substantially between common‐garden sites (Table 2, Fig. S2). These differences were most striking between Ephraim and Majors Flat, which are separated only by 8 km. Ephraim is located in a basin, whereas Majors Flat is located 415 m higher in the mountains. However, minimum temperatures were consistently lower at Ephraim (Table 1) as result of cold air drainage during the spring and summer and temperature inversions (i.e., “atmospheric decoupling”; Daly, Conklin, & Unsworth, 2010; Schuster, Kirchner, Jakobi, & Menzel, 2013) during the winter. Daily minimum temperatures during the winter in Ephraim were an average of 2.7°C and 6.8°C less than Majors Flat and Orchard, respectively. These differences were most pronounced during subregional temperature inversion events (settling and trapping of cold air within meters to km above the earth surface) where minimum temperatures were at most 18.6°C and 28.5°C less at Ephraim than Majors Flat and Orchard, respectively (Fig. S3). Another climatic factor that may have impacted survival is low snow depth. A large winter precipitation gradient occurs between basins and ranges, with a larger accumulation of snow in the mountains rather than the basins. We hypothesize that low minimum temperatures and shallow snow depth in Ephraim impacted the low survival in the Ephraim garden, especially in subspecies *vaseyana* (20% survival). The current study design does not allow us to specifically test for the presence of microclimate adaptations; we suggest that future work examine this. Differences in microclimate, namely freezing temperatures, have been known to be an important determinant in other desert species distribution (Franco & Nobel, 1989; Loik & Nobel, 1993; Shreve, 1911). Further, research in *A. tridentata* has found low survival in *vaseyana* in areas where a typically high snow pack was absent, yet high survival in areas with a snow cover present (Hanson, Johnson, & Wight, 1982). Further, Loik and Redar (2003) suggested that *A. tridentata* seedlings at higher elevations may have less exposure to freezing due to greater prevalence of snow. Consistent with this, Brabec et al. (2016) found that subspecies *vaseyana* had the least and *wyomingensis* had the greatest physiological avoidance and resistance to freezing among subspecies, and they proposed the seemingly ironic differences were due to greater insulating snow cover at higher elevation during winter and spring. These results suggest that selection for cold tolerance may relate to local‐scale microclimate variation, which we propose to be an important topic for further investigation.

Many traits can affect fitness, influencing growth and fecundity, along with survival. This study demonstrates moving warm‐adapted plants into cold climates decreases their survival. While there is little evidence for the inverse, low survival among cold‐adapted plants in a warm climate, trade‐offs in other fitness traits may exist. For example, seed yield data suggest that cold‐adapted plants had reduced fecundity in warmer climates compared to warm‐adapted plants (B.A. Richardson, unpublished data). Another possibility, which was beyond the scope of this experiment, is that cold‐adapted plants may have lower establishment success in warmer climates compared to warm‐adapted plants.

### 4.3 Applications

Future climate scenarios predict an increase in global mean temperatures and an increase in extreme weather patterns. As the climate that *A. tridentata* is adapted to is displaced, plants will need to migrate to new areas, a slow task for a sessile organism (Shaw & Etterson, 2012). The movement of plant species in response to rapid climatic warming will frequently be slower than phenotypic and adaptive genetic changes required to adjust to the novel climate. Due to population differentiation, the effects of climate change are likely to vary throughout a species range (Davis, Shaw, & Etterson, 2005). While locations subject to frequent cold air pooling are not likely to escape regionally increasing temperatures, they may act as microrefugia against the amplified temperature trends and variations (Dobrowski, 2011). Through genecological modeling of future climate scenarios, it is likely that as the climate changes, *A. tridentata* will not be genetically suited to the environment in which it currently grows, resulting in extirpation of some populations.

Movement of seed populations to areas outside of their adaptive breadth can have a negative impact on fitness (Hereford, 2009). Restoration of *A. tridentata* after fires or other disturbances has been a management priority over the past decade, yet success of these efforts has been varied (Arkle et al., 2014; Knutson et al., 2014). Planting *A. tridentata* seed outside their adaptive breadth could result in unsuccessful establishment and/or low fitness and provide an opportunity for invasive species encroachment leading to a loss of species diversity and ecosystem degradation (reviewed in Dumroese, Luna, Richardson, Kilkenny, & Runyon, 2015). Previous research has shown that sagebrush species/subspecies are adapted to different ecological niches such as elevation and soil type (McArthur, 1994). Moving sagebrush populations to different climatic or edaphic conditions is therefore not recommended (Mahalovich & McArthur, 2004). Our work is the first step to creating climate‐based guidelines for the transfer of seed for restoration purposes in *A. tridentata*. Being able to map traits relating to climate across the landscape is one practical advantage of genecology, which is particularly useful in highly heterogeneous environments such as western North America. This research will be the first step to delineate seed transfer zones, guidelines to ensure that seed used for restoration is adapted to the site.

## Data Achieving

Data for this study are available at the Dryad Digital Repository http://dx.doi.org/10.5061/dryad.32s2t
