# Supplementary material for: Climate drives adaptive genetic responses associated with survival in big sagebrush (Artemisia tridentata)
Source: Evol Appl. 2017 Mar 3;10(4):313–22. doi: 10.1111/eva.12440 (PMC5367076; doi:10.1111/eva.12440)
Supplement: Supplementary file 1 [file EVA-10-313-s001.pdf]

Supplementary Table 1. Population information from seed source location.

Population	Latitude	Longitude	Elev. (m)	Subspecies	Cytotype	Group	Ephraim (N)	Majors (N)	Orchard (N)
AZT1	35.917	-111.773	1898	tridentata	4	T4x	10	11	9
AZW1	35.825	-112.131	1914	tridentata	4	T4x	11	10	10
CAT1	40.541	-119.919	1213	tridentata	2	T2x	5	5	5
CAT2	37.797	-118.57	1974	tridentata	2	T2x	10	9	10
CAV2	37.723	-118.593	2330	vaseyana	2	V2x	10	10	8
CAV3	32.646	-116.186	827	vaseyana	4	V4x	7	7	5
CAV4	34.825	-118.872	1120	vaseyana	4	V4x	5	5	3
CAW3	40.6328	-120.6064	1884	vaseyana	2	V2x	9	9	5
COT1	39.613	-107.81	1790	tridentata	2	T2x	10	9	9
COW1	39.613	-107.81	1790	wyomingensis	4	W4x	10	9	10
COW2	40.181	-108.456	1788	wyomingensis	4	W4x	9	7	8
IDT1	43.096	-115.657	949	tridentata	2	T2x	9	9	10
IDT2	43.337	-116.008	939	tridentata	2	T2x	10	10	10
IDT3	43.337	-116.964	1358	tridentata	2	T2x	9	10	10
IDV2	43.678	-115.974	1003	vaseyana	2	V2x	6	7	4
IDV3	44.259	-114.64	1766	vaseyana	2	V2x	5	4	5
IDV4	44.508	-116.024	1438	vaseyana	2	V2x	2	2	2
IDV5	43.839	-116.256	1309	vaseyana	2	V2x	10	9	9
IDW1	43.093	-115.653	963	wyomingensis	4	W4x	9	9	9
IDW2	43.327	-116.004	977	wyomingensis	4	W4x	3	4	3
IDW3	43.466	-116.853	870	wyomingensis	4	W4x	10	10	10
MTT1	45.206	-108.826	1240	tridentata	2	T2x	6	6	6
MTV1	45.172	-108.455	2156	vaseyana	2	V2x	6	4	2
MTW1	45.655	-105.155	972	tridentata	2	T2x	1	1	1
MTW1	45.655	-105.155	972	wyomingensis	4	W4x	6	6	5
MTW2	46.322	-105.826	757	wyomingensis	4	W4x	9	9	9
MTW3	45.207	-108.783	1458	wyomingensis	4	W4x	10	9	10
NMT1	35.98	-107.226	2122	tridentata	4	T4x	10	10	8
NMT2	35.757	-107.148	1979	tridentata	4	T4x	10	10	10
NVT1	39.292	-117.852	1629	tridentata	2	T2x	10	10	10
NVT2	38.797	-115.392	1797	tridentata	2	T2x	10	9	10
NVV1	39.473	-117.05	2412	vaseyana	2	V2x	9	9	6
NVV1	39.473	-117.05	2412	vaseyana	4	V4x	1	1	1
NVV2	38.818	-115.284	2132	vaseyana	2	V2x	9	8	8
NVV2	38.818	-115.284	2132	vaseyana	4	V4x	1	1	1
NVV3	36.305	-115.617	2410	vaseyana	4	V4x	10	9	7
NVW1	41.515	-117.775	1349	tridentata	2	T2x	10	8	9
ORT1	42.975	-117.185	1349	tridentata	2	T2x	10	10	11
ORT2	45.759	-119.208	223	tridentata	2	T2x	9	10	11

ORT3	42.326	-119.367	1782	tridentata	2	T2x	10	11	10
ORV1	44.488	-117.323	986	vaseyana	4	V4x	9	10	10
ORV2	42.321	-119.354	1828	vaseyana	2	V2x	2	3	3
ORW1	43.785	-118.259	1196	wyomingensis	4	W4x	3	3	4
ORW2	42.317	-119.393	1707	tridentata	2	T2x	10	11	9
ORW3	44.134	-120.356	1048	tridentata	2	T2x	3	3	2
UTT1	37.993	-112.509	2106	tridentata	2	T2x	10	10	10
UTT2	38.306	-109.388	1820	tridentata	2	T2x	10	9	10
UTT3	37.04	-112.264	1618	tridentata	4	T4x	2	2	2
UTT3	37.04	-112.264	1618	tridentata	2	T2x	8	8	7
UTV1	39.341	-111.522	2097	vaseyana	2	V2x	5	3	5
UTV2	37.993	-112.509	2118	wyomingensis	4	W4x	10	10	10
UTV3	38.341	-109.217	2326	vaseyana	4	V4x	8	6	8
UTW1	38.328	-109.435	1797	wyomingensis	4	W4x	10	10	10
UTW2	41.8566	-113.0499	1438	wyomingensis	4	W4x	10	9	9
WAT1	46.7419	-118.2278	329	tridentata	2	T2x	10	10	10
WAT2	46.9488	-119.2371	312	tridentata	4	T4x	9	9	7
WAW1	46.7672	-119.4727	297	wyomingensis	4	W4x	8	7	8
WYW1	42.6006	-109.8341	2111	wyomingensis	4	W4x	2	1	2
WYW2	42.5923	-110.0854	2097	wyomingensis	4	W4x	4	2	3
Total							*449*	*432*	*418*

Geography, subspecies, and cytotype information from seed collections sites (populations) and sample size (N). Group is the subspecies by cytotype combination.

Supplementary Table 2. Names and definitions of climate used in genecology model. Source-population climate were obtained from thin plate splines.

Variable name	Definition
adi	Annual dryness index: sqrt(dd5)/map
adiminddd0	Annual dryness and cold index: (sqrt(dd5))/map * mmindd0
d100	Julian date the sum of degree-days >5 degrees C reaches 100
dd0	Degree-days <0 degrees C (based on mean monthly temperature)
dd0gsp	dd0 / gsp
dd0map	dd0 / map
dd5	Degree-days >5 degrees C (based on mean monthly temperature)
dd5mtcm	dd5 * mtcm
fday	Julian date of the first freezing date of autumn
ffp	Length of the frost-free period (days)
gsdd5	Degree-days >5 degrees C accumulating within the frost-free period
gsp	Growing season precipitation (April to September)
gspdd5	(gsp * dd5)/1000
gspmtcm	(gsp * mtcm)/1000
gsptd	(gsp * (mtwm - mtcm))/100
map	Mean annual precipitation
mapdd5	(map * dd5)/1000
mapmtcm	(map * mtcm)/1000
maptd	(map * (mtwm - mtcm))/100
mat	Mean annual temperature
mmax	Mean maximum temperature in the warmest month
mmin	Mean minimum temperature in the coldest month
mmindd0	mmindd0
mtcm	Mean temperature in the coldest month
mtcmgsp	mtcm/gsp
mtcmmap	mtcm/map
mtwm	Mean temperature in the warmest month
pratio	gsp/map
sday	Julian date of the last freezing date of spring
sdi	Summer dryness index: (sqrt(gsdd5))/gsp
sdimindd0	(sqrt(gsdd5)/gsp) * mmindd0
sdimtcm	(sqrt(gsdd5)/gsp) * mtcm
smrp	Summer precipitation: (July + August)
smrpb	Summer precipitation balance: (Jul+Aug+Sep)/(Apr+May+Jun)
smrsprpb	Summer/Spring precipitation balance: (Jul+Aug)/(Apr+May)
sprp	Spring precipitation: (Apr+May)
tdgsp	(mtwm - mtcm)/gsp
tdiff	Temperature difference: mtwm - mtcm
tdmap	(mtwm - mtcm)/map
winp	Winter precipitation: (Nov+Dec+Jan+Feb)

Note: Temperature-related variables are defined in units of degrees C and precipitation values in mm.

Supplementary Table 3. Analysis of big sagebrush by subspecies:cytotype group using regression for a parametric survival model (accelerated failure time).

	Value	*Standard Error*	*z*	*P*
T4x (Intercept)	3.945	0.099	39.942	0.000
T2x	-0.089	0.111	-0.796	0.426
W4x	-0.157	0.117	-1.333	0.182
V2x	-0.336	0.126	-2.676	0.007
V4x	-0.810	0.142	-5.707	0.000
Log(scale)	-0.433	0.046	-9.451	0.000
Scale = 0.649				

This model assumes that survival time changes by a constant factor when comparing different levels of covariates. T4x is the baseline (intercept) value. The coefficient value is the factor by which the survival time is multiplied for that covariate compared to the baseline. The scale indicates how the rate of survival changes over time. The *z*-values and *p*-values are used to test for statistical significance of any difference between groups. T4x = tetraploid *tridentata;* T2x = diploid *tridentata;* W4x = tetraploid *wyomingensia;* V2x = diploid *vaseyana;* V4x = tetraploid *vaseyana*

Supplementary Table 4. Probability of and median survival for each subspecies:cytotype group of big sagebrush.

Months	T4x	T2x	W4x	V2x	V4x
12	0.981<br>(0.944, 1.000)	0.982<br>(0.963, 1.000)	0.982<br>(0.958, 1.000)	0.959<br>(0.914, 1.000)	0.732<br>(0.608, 0.881)
24	0.923<br>(0.853, 0.998)	0.818<br>(0.762, 0.878)	0.735<br>(0.657, 0.821)	0.575<br>(0.472, 0.701)	0.342<br>(0.223, 0.522)
36	0.596<br>(0.477, 0.746)	0.594<br>(0.525, 0.673)	0.522<br>(0.438, 0.623)	0.452<br>(0.351, 0.538)	0.195<br>(0.105, 0.363)
48	0.481<br>(0.362, 0.638)	0.435<br>(0.367, 0.517)	0.460<br>(0.377, 0.562)	0.288<br>(0.201, 0.413)	0.098<br>(0.039, 0.248)
59	0.481<br>(0.362, 0.638)	0.406<br>(0.338, 0.487)	0.398<br>(0.317, 0.500)	0.260<br>(0.177, 0.383)	0.098<br>(0.039, 0.248)
Median	47<br>(35, NA)	47<br>(41, 51)	41<br>(29, 59)	35<br>(23, 47)	23<br>(23, 25)

Probability of survival shown for 12, 24, 36, 48 and 59 months and median survival shows at which month there is 50% chance of survival. Upper and lower 95% confidence intervals for each of the values are shown in parenthesis.

Supplementary Table 5. Probability of survival, median survival, and mortality numbers for each source-population of big sagebrush in the Ephraim common garden.

Population	12mo	24mo	36mo	48mo	59mo	Median	Dead	Total	Proportion Dead
MTT1	1.000	1.000	1.000	1.000	1.000	-	0	6	0.000
MTW2	1.000	1.000	1.000	1.000	1.000	-	0	9	0.000
NMT1	1.000	1.000	1.000	0.800	0.800	-	2	10	0.200
COW2	1.000	0.778	0.778	0.778	0.778	-	2	9	0.222
WAT2	1.000	1.000	0.778	0.778	0.778	-	2	9	0.222
MTW1	1.000	0.857	0.714	0.714	0.714	-	2	7	0.286
COT1	1.000	1.000	0.900	0.800	0.700	-	3	10	0.300
UTT2	0.900	0.800	0.700	0.700	0.700	-	3	10	0.300
WAT1	1.000	1.000	0.900	0.700	0.700	-	3	10	0.300
ORW1	1.000	0.667	0.667	0.667	0.667	-	1	3	0.333
IDV3	1.000	0.800	0.600	0.600	0.600	-	2	5	0.400
UTV1	1.000	0.800	0.600	0.600	0.600	-	2	5	0.400
IDT3	0.889	0.889	0.667	0.556	0.556	-	4	9	0.444
ORT2	1.000	0.778	0.667	0.556	0.556	-	4	9	0.444
MTV1	1.000	1.000	1.000	0.833	0.500	51	3	6	0.500
UTV2	1.000	1.000	0.900	0.800	0.500	49	5	10	0.500
WYW1	1.000	1.000	0.500	0.500	0.500	25	1	2	0.500
IDT2	1.000	0.600	0.500	0.500	0.400	42	6	10	0.600
NVT1	1.000	1.000	0.600	0.400	0.400	44	6	10	0.600
NVT2	1.000	1.000	0.600	0.500	0.400	43	6	10	0.600
UTT1	1.000	1.000	0.600	0.500	0.400	50	6	10	0.600
UTW1	1.000	0.900	0.600	0.500	0.400	45	6	10	0.600
WAW3	0.875	0.625	0.375	0.375	0.375	30.5	5	8	0.625
AZW1	0.909	0.909	0.364	0.364	0.364	35	7	11	0.636
IDT1	1.000	0.667	0.556	0.333	0.333	41	6	9	0.667
IDV2	0.833	0.833	0.667	0.333	0.333	39	4	6	0.667
IDW1	1.000	0.778	0.556	0.444	0.333	37	6	9	0.667
AZT1	1.000	0.900	0.600	0.300	0.300	39	7	10	0.700
COW1	0.900	0.600	0.300	0.300	0.300	26	7	10	0.700
MTW3	1.000	0.800	0.500	0.500	0.300	43	7	10	0.700
NMT2	1.000	0.800	0.300	0.300	0.300	27.5	7	10	0.700
ORT3	1.000	1.000	0.600	0.400	0.300	44	7	10	0.700
UTT3	0.900	0.800	0.500	0.300	0.300	38	7	10	0.700
WYW2	1.000	1.000	0.500	0.250	0.250	41	3	4	0.750
CAW3	0.889	0.556	0.333	0.222	0.222	29	7	9	0.778
ORV1	0.889	0.333	0.333	0.222	0.222	23	7	9	0.778
CAT1	1.000	0.600	0.200	0.200	0.200	25	4	5	0.800
IDV5	1.000	0.900	0.600	0.200	0.200	44	8	10	0.800
NVV1	1.000	0.500	0.500	0.200	0.200	32	8	10	0.800

NVV2	1.000	0.200	0.200	0.200	0.200	23	8	10	0.800
NVW1	1.000	0.600	0.600	0.200	0.200	44	8	10	0.800
ORT1	1.000	0.800	0.600	0.200	0.200	44	8	10	0.800
UTV3	0.875	0.625	0.250	0.125	0.125	27	7	8	0.875
NVV3	1.000	0.400	0.200	0.100	0.100	23	9	10	0.900
ORW2	1.000	0.500	0.300	0.100	0.100	24	9	10	0.900
CAT2	1.000	0.700	0.300	-	-	30.5	10	10	1.000
CAV2	0.900	-	-	-	-	23	10	10	1.000
CAV3	0.286	0.143	-	-	-	11	7	7	1.000
CAV4	0.200	-	-	-	-	11	5	5	1.000
IDV4	1.000	1.000	0.500	-	-	36	2	2	1.000
IDW2	1.000	0.333	-	-	-	23	3	3	1.000
IDW3	1.000	0.400	0.100	-	-	23	10	10	1.000
ORV2	1.000	0.500	0.500	-	-	32	2	2	1.000
ORW3	1.000	0.667	-	-	-	25	3	3	1.000
UTW2	1.000	0.400	0.200	-	-	23	10	10	1.000

Probability of survival is shown after 12, 24, 36, 48 and 59 months and median survival shows at which month there is 50% chance of survival. Number of dead and total number of plants. Table is sorted by proportion died per population.

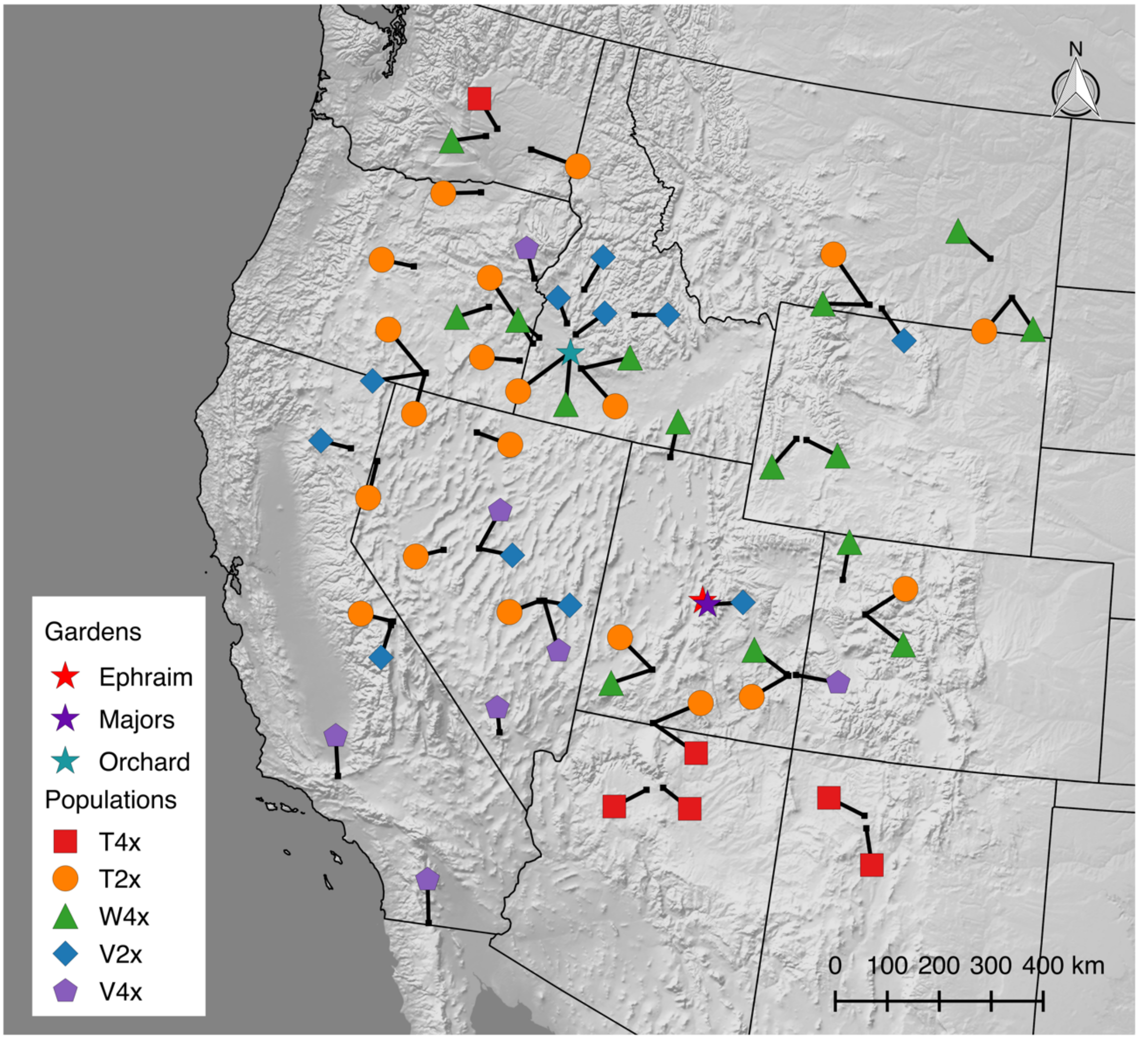

Supplementary Figure 1: Experimental design includes three common garden locations (stars) and 55 source populations (pentagons). Populations are seed source locations of three big sagebrush subspecies and two cytotype levels (indicated by colored polygons). T4x = tetraploid *tridentata;* T2x = diploid *tridentata;* W4x = tetraploid *wyomingensia;* V2x = diploid *vaseyana;* V4x = tetraploid *vaseyana*.

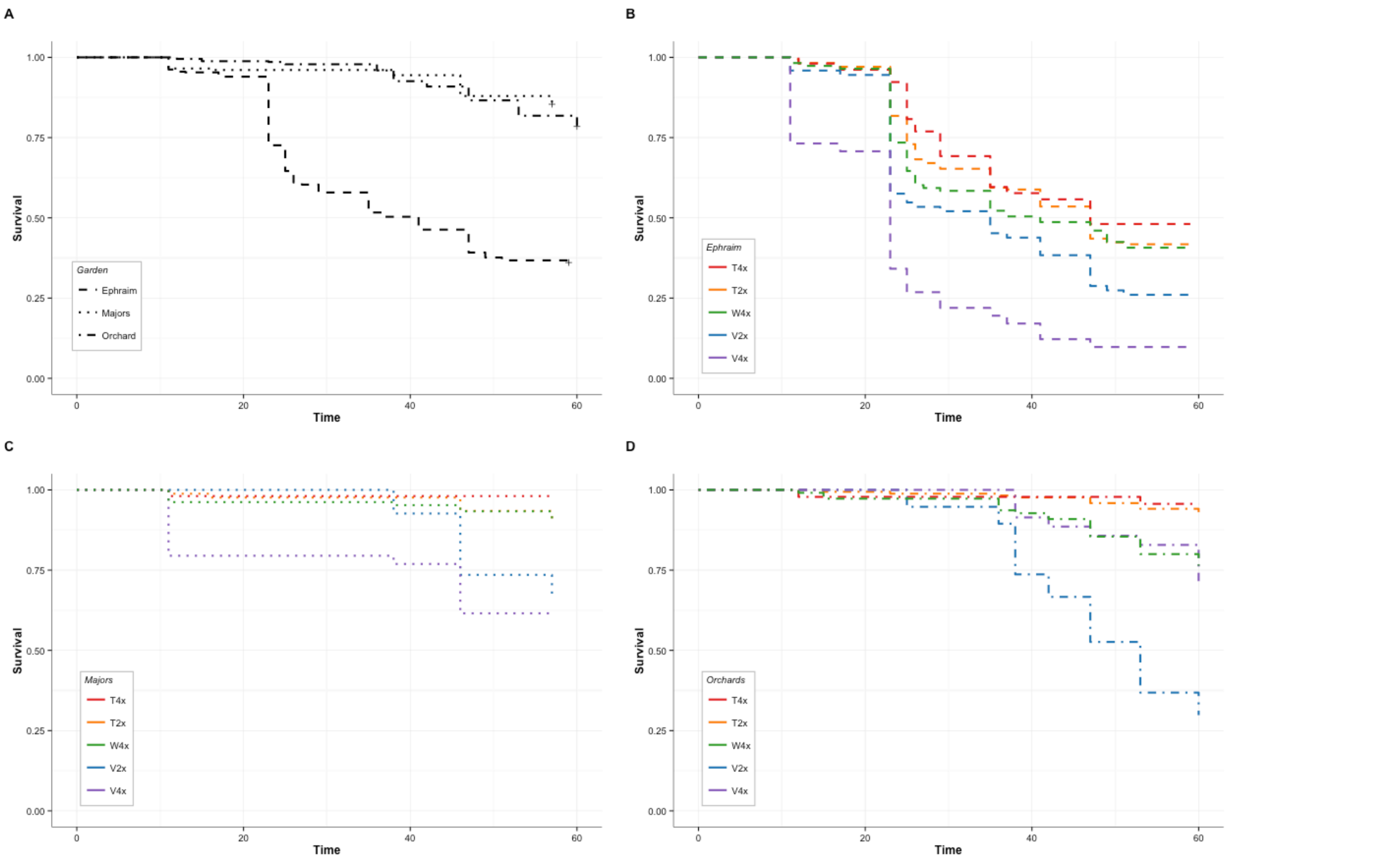

Supplementary Figure 2. Kaplan-Meier survivorship curves for big sagebrush by (a) common garden and survivor ship by subspecies:cytotype group for the (b) Ephraim, (c) Majors Flat and (d) Orchards garden.

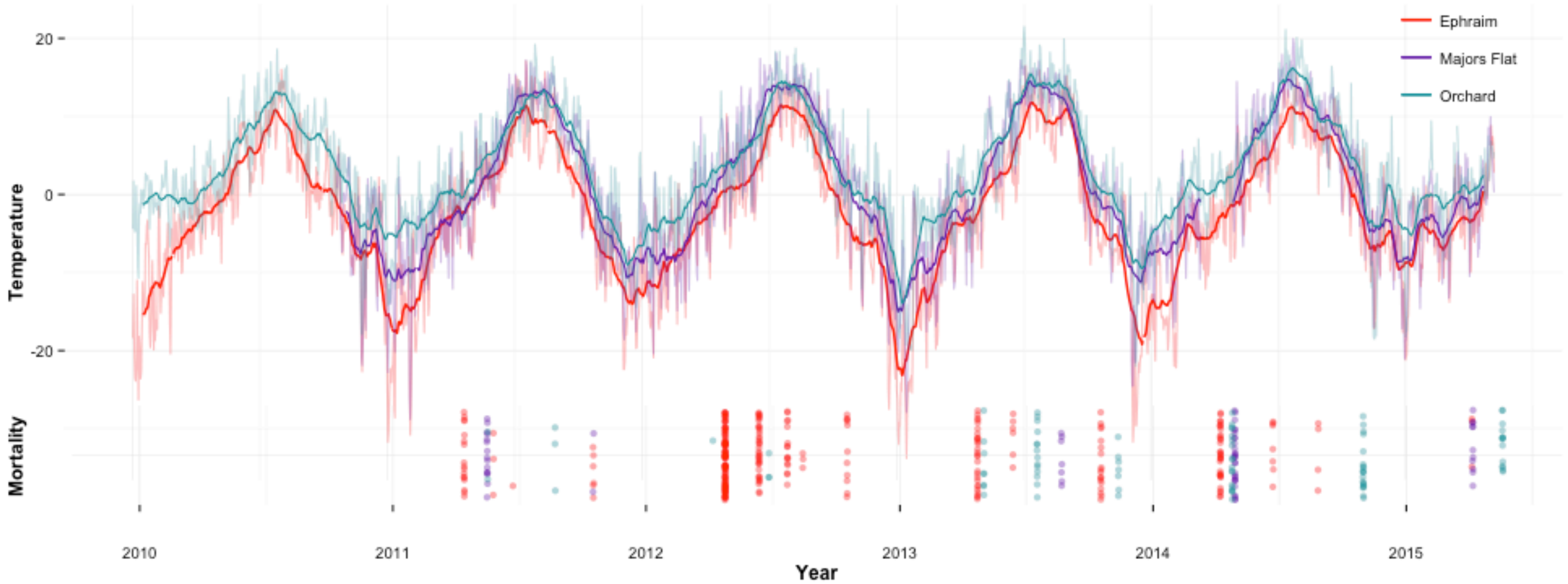

Supplementary Figure 3. Top panel shows minimum daily temperatures (light lines) with 30 day moving averages (bold lines) for the three garden locations. Bottom panel shows number of deaths for each garden at each census time. Ephraim (red), Majors Flat (purple) and Orchard (turquoise) garden.
